# Cellulose Conversion Into Hexitols and Glycols in Water: Recent Advances in Catalyst Development

**DOI:** 10.3389/fchem.2019.00834

**Published:** 2019-11-29

**Authors:** Oleg V. Manaenkov, Olga V. Kislitsa, Valentina G. Matveeva, Ester M. Sulman, Mikhail G. Sulman, Lyudmila M. Bronstein

**Affiliations:** ^1^Department of Biotechnology, Chemistry, and Standardization, Tver State Technical University, Tver, Russia; ^2^Regional Technological Center, Tver State University, Tver, Russia; ^3^A.N. Nesmeyanov Institute of Organoelement Compounds, Russian Academy of Sciences, Moscow, Russia; ^4^Department of Chemistry, Indiana University, Bloomington, IN, United States; ^5^Department of Physics, Faculty of Science, King Abdulaziz University, Jeddah, Saudi Arabia

**Keywords:** cellulose, hydrolytic hydrogenation, hydrogenolysis, sorbitol, mannitol, ethylene glycol, propylene glycol

## Abstract

Conversion of biomass cellulose to value-added chemicals and fuels is one of the most important advances of green chemistry stimulated by needs of industry. Here we discuss modern trends in the development of catalysts for two processes of cellulose conversion: (i) hydrolytic hydrogenation with the formation of hexitols and (ii) hydrogenolysis, leading to glycols. The promising strategies include the use of subcritical water which facilitates hydrolysis, bifunctional catalysts which catalyze not only hydrogenation, but also hydrolysis, retro-aldol condensation, and isomerization, and pretreatment (milling) of cellulose together with catalysts to allow an intimate contact between the reaction components. An important development is the replacement of noble metals in the catalysts with earth-abundant metals, bringing down the catalyst costs, and improving the environmental impact.

## Introduction

The recent data show that around 1.1·10^11^ tons of lignocellulose biomass (including both lignin and cellulose) are synthesized in Nature annually, the majority of which (30–55%) is cellulose (Li et al., 2018a). Cellulose is a natural polymer consisting of glucose repeating units and is the most promising alternative for syntheses of value-added chemicals to replace non-renewable resources such as gas and oil (Li et al., 2015b, 2018a). Currently, there are numerous methods for direct catalytic conversion of cellulose to a number of valuable chemicals such as glucose (Shrotri et al., 2016, 2018), hexitols (Li et al., 2015a; Zada et al., 2017; Shrotri et al., 2018), glycols (Li et al., 2015a; Zheng et al., 2017), 5-hydroxymethylfurfural (Li et al., 2018b), methane (Wang et al., 2018), hydrogen (Wen et al., 2010), hexane and hexanols (Liu et al., 2014, 2015; Op De Beeck et al., 2015).

**Figure 1:**
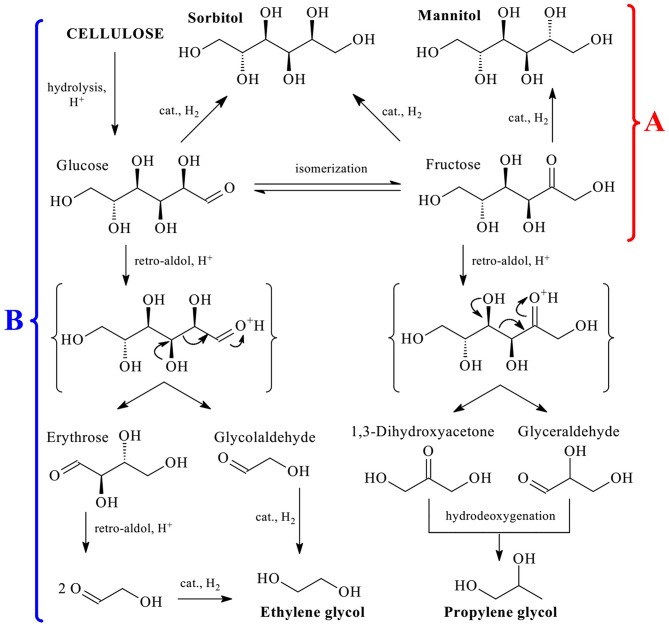
Schematic representation of hydrolytic hydrogenation **(A)** and hydrogenolysis **(B)** of cellulose to hexitols and glycols, respectively.

The presence of a large number of hydroxyl groups in the cellulose structure determines the optimal route of its conversion to polyols via hydrolytic hydrogenation or hydrogenolysis (involving C-C cleavage) (Li et al., 2015a). Both processes consist of several reactions but can be carried out in a one-pot procedure. Major products of hydrolytic hydrogenation are hexitols (sorbitol and mannitol, Figure 1A), while in hydrogenolysis they are glycols (ethylene glycol, EG, and propylene glycol, PG, Figure 1B). From the viewpoint of green chemistry, the best reaction medium for these processes is subcritical water (Rinaldi, 2014; Shitu et al., 2015) due to its availability, low cost, and non-toxicity. In subcritical water the concentration of both hydroxyls and hydroxonium ions increases by a factor of 35 (compared to r.t. water), which leads to acceleration of the reactions catalyzed by acids or bases, for example, hydrolysis (Gagic et al., 2018; Abaide et al., 2019). Moreover, the efficiency of both processes are strongly dependent on the hydrogenation catalyst activity, i.e., its ability to rapidly and selectively hydrogenate hexoses and induce their decomposition following retro-aldol mechanism.

In this review we discuss most interesting and promising catalytic systems which were proposed for hydrolytic hydrogenation and hydrogenolysis of cellulose in hydrothermal conditions in water in the last 5 years.

## Cellulose Hydrolytic Hydrogenation

As was demonstrated in a recent review (Makhubela and Darkwa, 2018), among noble metals Ru is the most active and employed in the majority of catalytic systems proposed for hydrolytic hydrogenation of cellulose (Li et al., 2015a; Ribeiro et al., 2015b). In studies of Ru-containing catalysts, the major focus is on various supports and attempts to increase the catalyst efficiency via control of the support structure and properties.

### Carbon Based Supports

Carbon based materials are popular catalyst supports for cellulose conversion due to high surface area, porosity, and mechanical and thermal stability (Ribeiro et al., 2017a; Adsuar-Garcia et al., 2018). The Ru-containing catalyst based on commercial carbon black treated with H_2_SO_4_ to create acidic surface sites displayed comparatively high selectivity to sorbitol (40% at 190°C, 5 MPa, 3 h) and stability upon repeated use (Adsuar-Garcia et al., 2018). Ribeiro et al. compared Ru-containing catalysts based on original multiwall carbon nanotubes (CNT) and those modified by HNO_3_ to impart oxygenated groups (Ribeiro et al., 2017b). Although the activity of the catalyst based on original CNT was lower than that for the modified support (due to increased acidity), the maximum selectivity to sorbitol reached 70% (205°C, 5 MPa, 2 h), while for the modified support, it was lower by 20%. Clearly, acidity is important for cellulose hydrolysis, but it should be moderate to maintain a high sorbitol yield. This catalyst showed excellent stability upon four reuses. Micro/mesoporous activated carbon containing sulfonic groups and Pt nanoparticles allowed a total hexitol yield of 69.5% at comparatively mild conditions (180°C, 2 MPa, 24 h) (Lazaridis et al., 2017). It is noteworthy, that Ru on the same support showed a much lower hexitol yield, but allowed for high yields of glycerol and PG due to increased hydrogenolysis of hexitols formed. Both catalysts were stable upon four consecutive reaction, while a decreased number of sulfonic groups did not influence the activity and selectivity to hexitols. Rey-Raap et al. reported glucose-derived carbons obtained in the presence of CNT as a support for Ru nanoparticles (Rey-Raap et al., 2019). These materials possess high surface areas, abundant microporosity and low acidity, which increase Ru dispersion and the sorbitol yield to 64.1% (205°C, 5 MPa, 2 h). This type of catalysts is also cost effective as a significant fraction of CNT is replaced with low-cost biomass generated carbon as well as stable. No deterioration of the cellulose conversion or sorbitol yield was observed after four consecutive reactions, which the authors assign to the unchanged Ru oxidation state (according to XPS). A similar approach to low-cost supports was utilized by Li et al. (2019). Carbonized cassava dregs (formed in the starch production) functionalized with SO_3_H groups and containing Ru nanoparticles allowed for a comparable sorbitol yield (63.8%) at 180°C, 4 MPa for 10 h. The catalyst was stable in hydrothermal conditions in at least five consecutive cycles. Ru-Ni bimetallic catalysts based on activated carbon or CNT (Ru-Ni/AC; Ru-Ni/CNT) have been proposed for direct cellulose conversion to sorbitol (Ribeiro et al., 2017c). Independently of the support, Ni in bimetallic nanoparticles had a promoting effect on catalysis, increasing conversion, and selectivity to sorbitol due to intimate interaction of both metals. The sorbitol yield was in the range of 50–60% (205°C, 5 MPa, 5 h), but it could be further increased to more than 70% when cellulose and catalysts were milled together, an important development in the catalyst pretreatment (Ribeiro et al., 2015a; Liu et al., 2016b). These catalysts preserved their activity in at least four consecutive reactions.

Matveeva et al. reported Ru-containing catalysts based on micro/mesoporous hypercrosslinked polystyrene (HPS), allowing for the sorbitol yield of ~ 50% (245°C, 6 MPa, 0.08 h) at cellulose conversion of 85% (Matveeva et al., 2017). High efficiency of the catalyst is believed to be due to the HPS high surface area and a narrow pore size distribution, controlling the formation of 1.4 ± 0.3 nm Ru-containing nanoparticles. The catalyst stability in at least three catalytic cycles was demonstrated.

### Metal Oxide Based Supports

The catalyst based on CuO (a catalytic phase) and the CeO_2_-ZrO_2_ support has showed exceptional efficiency: 99.1% selectivity to sorbitol at 92% of the cellulose conversion (245°C, 5.2 MPa, 4 h) in the neutral medium (Dar et al., 2015). Moreover, the catalyst was stable during five consecutive catalytic reactions. The authors believe that such outstanding properties should be attributed to the mesoporous structure of the support, facilitating mass transfer as well as enhanced ability of the Cu-containing catalyst in promoting hydrogenation.

Ru-containing catalysts based on zeolites were also successfully utilized. Wang et al. obtained 39.4% yield of hexitols using mesoporous Ru/HZSM-5 at 200°C, 5 MPa for 10 h (Wang et al., 2015b). The authors believe that Lewis (LAS) and Brønsted (BAS) acid sites formed during the zeolite treatment promote cellulose hydrolysis. In addition, the mesoporous structure of the support increases the surface area and provides better Ru-containing nanoparticle dispersion, leading to a comparatively high yield of hexitols. A significantly higher yield (up to 63.6%) has been obtained with Ni-containing catalysts based on ZSM-5 (Liang et al., 2015). Formation of electron deficient multiply-twinned Ni particles imbedded in zeolite allows for excellent CO adsorption and activation of glucose carbonyl groups, facilitating its hydrogenation with active hydrogen. This is an especially remarkable development because a noble metal (Ru) was replaced with an earth-abundant metal, leading to a greener and cheaper catalyst. Approximately similar hexitol yield (60%) was obtained for Ni/mesoporous-ZSM-5 (240°C, 4 MPa, 2.5 h) (Zhang et al., 2016). Here, the ZSM-5 synthesis was carried out using nanocrystalline cellulose as a template, creating mesopores in a simple and inexpensive way. However, the authors recognize that there is room for improvement as the catalyst is unstable in hydrothermal conditions, with a decreasing hexitol yield to 30 and 10% in the second and third uses, respectively. That is believed to be a consequence of the damage of the zeolite mesoporous structure and aggregation of Ni nanoparticles.

## Cellulose Hydrogenolysis

There is a high demand for EG and PG for various applications. In 2015, 23 million tons of EG and 1.8 million tons of PG were produced, while the growth prognosis for these compounds for the next 10–20 years is on average 5% a year (Pang et al., 2016; Zheng et al., 2017). However, cellulose conversion to EG and PG is very complicated (Figure 1B) and includes several reactions: hydrolysis, isomerization, retro-aldol condensation, hydrogenation, and hydrodeoxygenation (Rinaldi, 2014; Zheng et al., 2017). In this respect, the development of highly selective catalytic systems for this process is an important and challenging task.

### W-Containing Catalysts

Recent studies showed that the high yield of glycols in cellulose hydrogenolysis occurs in the presence of catalysts containing tungsten compounds (Table 1) (Zheng et al., 2017). W-containing catalysts can be used in conjunction with the hydrogenation catalysts or bifunctional catalysts including the hydrogenation catalyst in the W-containing phase can be formed.

**Table 1:** Catalytic properties of W-containing catalysts in conversion of cellulose to glycols^a^.

**Catalysts**	**Reaction conditions (substrate, temperature, pressure, time)**	***X*, %**	**η_**EG**_ (*S*_**EG**_), %**	**η_**PG**_ (*S*_**PG**_), %**	**References**
W(0.24)-MA + Ru/C (5%)	MCC; 518 K; 6 MPa H_2_; 0.5 h	43.5	*S*_EG_ 19.4	*S*_PG_ 3.6	Wang et al., 2015a
6 wt.% WO_3_/C + 3 mas.% Ru/C	MCC; 478 K; 6 MPa H_2_; 0.5 h	~ 62	*S*_EG_ 55.8	*S*_PG_ 12.3	Liu and Liu, 2016
1% Ru/WO_3_	MCC; 240°C; 4 MPa H_2_; 2 h	100	η_EG_ 76.3	η_PG_ 4.3	Li et al., 2017
1% Ru/h-WO_3_	MCC; 240°C; 4 MPa H_2_; 2 h	100	η_EG_ 77.5	η_PG_ 4.4	Li et al., 2018b
W/Zr + Ru/C	MCC; 215°C; 5.2 MPa H_2_; 1.5 h	90	η_EG_ 58.8	η_PG_ 4.5	Chai et al., 2017
0.8% Ru-30% W/CNT	BMC; 205°C; 50 bar H_2_; 3 h	100	η_EG_ 40.0	η_PG_ 7.2	Ribeiro et al., 2018a
5% Ru-30% W_18_O_40_/graphene	MCC; 245°C; 6 MPa H_2_; 1 h	100	η_EG_ 62.5	η_PG_ 5.1	Zhang et al., 2019
Ru/CG_HNO3_ + W/CG	BMC; 205°C; 50 bar H_2_; 5 h	100	η_EG_ 48.4	η_PG_ 0.0	Ribeiro et al., 2019
10% Ni-20% W/SBA-15 (1.00)	MCC; 518 K; 5 MPa H_2_; 2 h	100	η_EG_ 64.9	η_PG_ 6.5	Xiao et al., 2017b
5% Ni-20% W/SiO_2_	MCC; 240°C; 5 MPa H_2_; 2 h	100	η_EG_ 63.3	η_PG_ 4.7	Xiao et al., 2018a
15% Ni-20% W/SiO_2_-OH	MCC; 240°C; 5 MPa H_2_; 2 h	100	η_EG_ 63.1	η_PG_ 1.9	Xiao et al., 2018c
3Ni-15W-3Al	MCC; 503 K; 4 MPa H_2_; 1.5 h	100	*S*_EG_ 76.0	*S*_PG_ 8.3	Hamdy et al., 2017
30% Cu-30% WO_x_/AC + Ni/AC	MCC; 518 K; 4 MPa H_2_; 2 h	100	η_EG_ 70.5	η_PG_ 4.5	Chu and Zhao, 2018
30% NiWB (1:1)/CNTs	MCC; 523 K; 6 MPa; 2 h	100	η_EG_ 57.7	η_PG_ 4.6	Liu et al., 2016a
Ni_0.3_-W_0.3_/CNF	MCC; 245°C; 6 MPa H_2_; 2 h	95	η_EG_ 33.6	η_PG_ 7.1	Yang et al., 2016

a *X is the cellulose conversion; η_EG_ and η_PG_ are the yields of EG and PG, respectively; S_EG_ and S_PG_ are selectivities to EG and PG, respectively; MCC is microcrystalline cellulose; BMC is ball-milled cellulose; CG is glucose based carbon; SBA-15 is ordered silica; AC is activated carbon; CNF is carbon nanofibers, while subscript for Ni and W is weight percentage of each species*.

Wang et al. synthesized the W-containing catalyst based on ordered mesoporous alumina (MA) which was used together with Ru/C (5 wt.%) in cellulose hydrogenolysis (Wang et al., 2015a). The addition of W(*x*)-MA (*x* is the W fraction) allowed for the EG selectivity increase from 3.2 to 19.4%, while conversion remained the same. The authors concluded that W(*x*)-MA does not participate in cellulose depolymerization (although it contains both LAS and BAS), but influences the final distribution of the reaction products. A more detailed study on the distribution of hydrogenolysis products was presented in (Liu and Liu, 2016). The process was carried out with a mixture of Ru/C and WO_3_/C (6%) and the dependence of the product distribution on the catalyst was elucidated using kinetic studies of the three competitive reactions of glucose: hydrogenation, isomerization, and C–C bond cleavage. WO_3_ (solid acid) was found to stimulate cellulose hydrolysis and also to promote cleavage of the C–C bonds in C_6_ sugars, resulting in EG and PG instead of the sugar hydrogenation to corresponding hexitols on Ru/C. The carbon support with basic properties catalyzed isomerization of glucose to fructose, which again led to the preferential formation of EG and PG. The authors determined that this kinetic analysis allows one to forecast the maximum selectivity ratio of PG to EG (2.5) with the maximum PG yield of ~ 71% (Table 1). Li et al. synthesized Ru-containing catalysts based on WO_3_ nanocrystals of different shapes: rectangular nanosheets (Li et al., 2017), hexagonal nanorods (h-WO_3_), and monoclinic nanosheets (m-WO_3_) (Li et al., 2018b). Detailed structural studies showed the crucial importance of surface LAS formed upon adsorption of water on the surface of WO_3_ for cellulose conversion to EG. The catalyst based on h-WO_3_ crystals and containing the largest number of LAS allows for the highest EG yield (77.5%) (Table 1). A 5-fold catalyst reuse did not affect its activity, which could be explained by the unchanged WO_3_ structure and Ru dispersion. Similar conclusions were obtained by Chai et al. (2017), who compared a series of catalysts based on WO_3_-ZrO_2_ (WZr) in conjunction with Ru/C for one-pot cellulose conversion. It was demonstrated that W^5+^-OH species on the surface of WO_3_ are active catalytic sites for cleavage of the C_2_-C_3_ bond in glucose, while glycolaldehyde formed is hydrogenated to EG on the Ru/C surface.

Ribeiro et al. synthesized Ru- and W-containing mono- and bimetallic catalysts on CNT (Ribeiro et al., 2018a). Incorporation of tungsten in the catalyst along with Ru species shifted the reaction to EG (Table 1). The authors demonstrated a synergetic effect when both Ru and W species were present, while the EG yield could be controlled by the weight fraction of metals. Four repeated uses of the catalyst did not decrease its activity. The authors demonstrated that the structure of the catalyst surface remains unchanged and there is no W leaching, explaining the stability of the catalytic performance. In the other publication reduced graphene oxide (called graphene) has been utilized for syntheses of Ru, WO_3_, and Ru-W_18_O_49_ containing catalysts in the one-pot solvothermal synthesis, allowing reduction of Ru ions and graphene oxide as well as the formation of W_18_O_49_ nanowires (Zhang et al., 2019). The bimetallic catalyst showed the highest EG yield at the 100% cellulose conversion and stability in three consecutive cycles, after which the EG yield decreased due to dissolution of the tungsten species in the reaction solution. The catalyst support based on the glucose-derived carbons utilized for hydrolytic hydrogenation of cellulose (Rey-Raap et al., 2019) has been also proposed for cellulose hydrogenolysis (Table 1) (Ribeiro et al., 2019). In this case, the authors demonstrated similar EG yields as those reported for CNT based catalysts (Ribeiro et al., 2018a), but with the much cheaper support at the similar catalyst stability.

As was discussed above, a replacement of noble metals with earth-abundant alternatives has a huge impact on process sustainability and cost. Xiao et al. synthesized multimetallic catalysts based on mesoporous silica (SBA-15) with the composition M-W/SBA-15 (M = Ni, Pd, Zn, Cu) (Xiao et al., 2017b) as well as on silica nanospheres SiO_2_-OH (Ni-W/SiO_2_) (Xiao et al., 2018a,c) and demonstrated good EG yields in the range 40–65% (Table 1). An exceptionally high EG selectivity (76%) at the 100% cellulose conversion has been achieved using the 3Ni-15W-3Al catalyst based on mesoporous siliceous material (Hamdy et al., 2017). The catalyst was synthesized in a one-pot procedure to incorporate isolated Al^3+^ ions, WO_3_ nanoparticles, and Ni^0^ nanoparticles with different loadings. The downside of this system is low catalyst stability as leaching of W and Ni after the catalytic reaction was observed (16% of W and 7% of Ni). Nevertheless, we believe this system deserves recognition and further improvement to eliminate leaching. Chu et al. synthesized Cu^0^-WO_x_ (2<x<3)/AC which allowed >70% EG yield when combined with Ni/AC (Table 1) (Chu and Zhao, 2018). A combination of Cu^0^ with WO_x_ nanoparticles leads to improved adsorption of a C_6_ sugar intermediate, followed by its conversion to C_2_-hydroxyaldehyde further hydrogenated to EG on the Ni species. It was determined that to balance C-C bond cleavage and hydrogenation, a molar ratio of Ni and W should be in the range 1/5–1/3.

CNT and CNF were also utilized as supports for Ni-W-containing catalysts (Table 1) (Liu et al., 2016a; Yang et al., 2016). Both catalysts showed good stability in two-three cycles and an abrupt decrease of the EG yield in further reactions. However, in the case of Ni_0.3_-W_0.3_/CNF, the authors determined that the activity loss is not due to leaching or the nanoparticle size change or carbon deposition, but solely due to retention of reaction products which shield active sites. Thus, the catalyst was easily regenerated with full restoration of its initial activity using simple purging with N_2_ for 30 min at 300°C. This makes the Ni_0.3_-W_0.3_/CNF catalyst promising for practical applications despite comparatively low EG yield.

It is noteworthy that the interaction of two types of catalytic species, acidic, and metallic in cellulose hydrogenolysis, is complex and not yet fully understood because of the combination of several processes (see the discussion above). On one hand, it was demonstrated that the reactions of the glucose retro-aldol condensation to glycolaldehyde and its hydrogenation to EG occur independently on different catalytic sites, thus, their intimate contact is not needed (Wiesfeld et al., 2019). On the other hand, the interaction of WO_3_ and metallic nanoparticles occurring at the electronic level leads to increase of the number of W^5+^ active sites (Li et al., 2017, 2018b; Chu and Zhao, 2018), which selectively break the C2-C3 bond in glucose (Chai et al., 2017). These data demonstrate the necessity of close contact between acidic and metallic sites. These inconsistencies show that more studies are needed for better understanding of the mechanism of these processes.

### Sn- and Co-containing Catalysts

Sun et al. proposed a catalytic system, selectivity of which could be switched from one diol to the other (Sun et al., 2016) using Sn species with different oxidation states in conjunction with Ni catalysts. In the case of combination of Ni/AC with a metallic Sn powder, the main hydrogenolysis product was EG with the yield of 57.6% (245°C, 5 MPa, 1.6 h). When a mixture of Ni/AC and SnO was used, PG with the yield of 32.2% (22.9% for EG) was formed. The authors demonstrated that the Sn species in the NiSn alloy formed *in situ* from Ni and Sn powders are active sites for the EG formation. It is noteworthy that both Sn in the alloy and SnO catalyze the glucose retro-aldol condensation to glycolaldehyde, but only SnO is active in the isomerization of glucose to fructose. These catalysts showed a relative stability in hydrothermal conditions, which according to authors' opinion make them promising for industrial applications. Xiao et al. observed a similar effect of the increasing PG yield upon using nano-Sn species with different oxidation states in combination with Ni catalysts (10%Ni−15%Sn/SBA-15) (Xiao et al., 2017a). Excellent glycol yields (55.2% for EG and 33.9% for PG) were obtained with the weakly basic Co/CeO_x_ catalyst (Li et al., 2019). The authors demonstrated that the interaction between well-dispersed Co species and the CeO_x_ support results in the formation of the Co^n+^-O_x_-Ce^3+^ base-acid pairs—main catalytic sites—providing the optimal balance between hydrolysis, retro-aldol condensation, isomerization, and hydrogenation and leading to high diol yields. Unfortunately, the lack of the catalyst stability studies does not allow one to evaluate the promise of these catalysts.

### Magnetically Recoverable Catalysts

Magnetically recoverable catalysts received considerable attention in all fields of catalysis including cellulose conversion due to economic and environmental benefits upon easy magnetic separation of catalysts (Li et al., 2015a; Liu and Zhang, 2016; Sudarsanam et al., 2018). To the best of our knowledge, direct conversion of cellulose to EG and PG with magnetically recoverable catalysts was reported only in (Manaenkov et al., 2016), although the utilization of magnetically recoverable catalysts in cellulose hydrolysis (Zhang and Fang, 2012; Li et al., 2015a), direct conversion of cellulose to sorbitol (Kobayashi et al., 2014; Zhang et al., 2014) and hydrogenolysis of sorbitol to glycols (Ye et al., 2012) has been previously described. The authors developed magnetically recoverable catalysts based on Ru-containing nanoparticles formed in the pores of magnetic silica, Fe_3_O_4_-SiO_2_. The highest selectivities to EG and PG were 19 and 20%, respectively, at 100% cellulose conversion (255°C, 6 MPa, 0.83 h) with the addition of 0.195 mol of Ca(OH)_2_ per 1 mol of cellulose. High stability in at least three consecutive cycles and easy magnetic separation from the reaction medium make this catalyst promising for applications in biomass conversion.

## Summary and Outlook

In summary, we can identify several major trends in hydrolytic hydrogenation and hydrogenolysis of cellulose:

Cellulose is converted in pure subcritical water, which is cheap, non-toxic and an excellent medium for acid-based catalyzed reactions;

Mineral acids as co-catalysts are replaced by simultaneous ball milling of the catalyst and cellulose to facilitate the process;

The majority of the proposed catalytic systems are bifunctional and could catalyze not only hydrogenation, but also hydrolysis, retro-aldol condensation, and isomerization, giving the best yields of target polyols—a strategy based on a balance of the catalytic efficiency toward the above reactions;

In the most promising catalytic systems, noble metals (Ru, Pt, etc.) are substituted by earth-abundant metals (Ni, Cu, Sn, Co, etc.), although the problem of stability of these systems still needs to be addressed.

Despite some unresolved issues, many reported catalysts possess excellent catalytic properties and stability in hydrothermal conditions, which allowed their successful testing not only in the conversion of pure microcrystalline cellulose, but also for cellulose containing materials such as cotton, cotton wool, paper (Ribeiro et al., 2017a,d, 2018b), wood lignocellulosic biomass (Yamaguchi et al., 2016; Ribeiro et al., 2018b; Xiao et al., 2018b; Pang et al., 2019), corn stalks, millet, sugarcane, etc. (Liu et al., 2017; Li et al., 2018a), which demonstrates a promising outlook for the future development of multi tonnage production of value-added chemicals and fuels from cellulose biomass.

## Author Contributions

All authors listed have made a substantial, direct and intellectual contribution to the work, and approved it for publication.

### Conflict of Interest

The authors declare that the research was conducted in the absence of any commercial or financial relationships that could be construed as a potential conflict of interest.
